# Effects of Exogenous Application of Glycine Betaine Treatment on ‘Huangguoggan’ Fruit during Postharvest Storage

**DOI:** 10.3390/ijms241814316

**Published:** 2023-09-20

**Authors:** Zhendong Zheng, Tie Wang, Miaoyi Liu, Xiaozhu Xu, Jun Wang, Guochao Sun, Siya He, Ling Liao, Bo Xiong, Xun Wang, Jiaxian He, Zhihui Wang, Mingfei Zhang

**Affiliations:** College of Horticulture, Sichuan Agricultural University, Chengdu 611130, China; zzd530362810@163.com (Z.Z.);

**Keywords:** antioxidant compounds, citrus, fruit quality, glycine betaine, postharvest, transcriptome analysis

## Abstract

Loss of quality in citrus fruit is a common occurrence during postharvest storage due to oxidative stress and energy consumption. In recent years, glycine betaine (GB) has been widely applied to postharvest horticulture fruit. This study aimed to investigate the effect of GB treatment (10 mM and 20 mM) on the quality and antioxidant activity of ‘Huangguogan’ fruit during postharvest storage at room temperature. Our results indicated that both 10 mM and 20 mM treatments effectively reduced weight and firmness losses and maintained total soluble solid (TSS), titratable acidity (TA), and ascorbic acid contents. Additionally, GB treatment significantly increased the activity of antioxidant enzymes, maintained higher levels of total phenols and total flavonoids, and led to slower accumulation of H_2_O_2_. A transcriptome analysis conducted at 28 days after treatment (DAT)identified 391 differentially expressed genes (DEGs) between 20 mM GB (GB-2) and the control (CK) group. These DEGs were enriched in various pathways, particularly related to oxygen oxidoreductase, peroxidase activity, and flavonoid biosynthesis. Overall, the application of GB proved beneficial in enhancing the storability and extending the shelf life of ‘Huangguogan’ fruit.

## 1. Introduction

The popularity of citrus among consumers is owed to its high contents of dietary fiber, carbohydrates, proteins, and minerals [1]. Tangor (*Citrus reticulata × Citrus sinensis*) is a significant category of citrus [2]. However, the high moisture content of tangor makes it susceptible to quality deterioration and flavor loss during postharvest storage due to physiological disorders. Consequently, there have been numerous attempts to enhance the physiological quality and prolong the shelf life of tangor fruit. Currently, postharvest citrus production methods primarily revolve around physical treatments, biocontrol agents, edible coatings, and emerging chemical strategies [3]. Chemical preservation has gained popularity as the preferred method due to its low cost, high efficiency, and ease of implementation [4,5]. Furthermore, natural inorganic and organic compounds, along with plant growth regulators, are widely utilized in postharvest production owing to their lack of residue and harmlessness to humans [6]. Nowadays, exogenous application of plant growth regulators, such as melatonin [7,8,9], jasmonic acid [10], and other plant hormones, has been employed to maintain bioactive compounds and enhance storability [11].

Among these bioactive substances, glycine betaine (GB) is classified as an alkaloid belonging to the quaternary ammonium base, exhibiting chemical similarities to amino acids. Farhang et al. [12] reported that hawthorn fruit treated with GB maintained a high level of ascorbic acid and increased the accumulation of anthocyanin. Additionally, application of GB delayed the degradation of titratable acid (TA) and firmness loss of ‘Hindi-Besennara’ mangoes [13]. A study on winter jujube has indicated that GB regulated the expression of energy-related enzyme genes, resulting in increasing the energy supply during storage. Consequently, more energy was allocated to the antioxidant system to counteract reactive oxygen species (ROS) [14]. GB has also been proven effective in alleviating chilling injuries in various fruits such as ‘Nanguo’ pears [15,16] and loquat fruit [17]. In addition, it enhanced the antioxidant capacity by increasing the activity of superoxide dismutase (SOD), catalase (CAT), and ascorbate peroxidase (APX). Moreover, GB improved the cold tolerance of peaches by modulating the energy state, membrane fatty acid metabolism, and production of various compounds [18]. The research of Roghayeh et al. [19] highlighted that GB treatment protected fruits from lipid peroxidation by scavenging ROS, thereby preserving fruit quality. These findings collectively suggest that GB treatment has significantly beneficial effects on fruit quality and holds promise as a potential method for regulating the physicochemical properties during postharvest stages.

‘Huangguogan’ fruit is a tangor variety that matures late [20] and is rich in beneficial bioactive compounds, particularly organic acids and antioxidant substances [21]. As the fruit undergoes the storage process, it gradually ages, resulting in the degradation of organic acids and a decrease in antioxidant activity [22]. However, there are limited reports on postharvest preservation technologies for ‘Huangguogan’ fruit. In order to enrich the postharvest preservation strategy of this variety, in this study, exogenous GB was applied to ‘Huangguogan’ fruit to assess its effect on fruit quality and antioxidant defense, revealing the enrichment of differentially expressed genes (DEGs) in ‘Huangguogan’ fruit after GB treatment. The discoveries presented in this study offer a fundamental and informative framework for the effective preservation of ‘Huangguogan’ during the postharvest period.

## 2. Results

### 2.1. Phenotype, Weight Loss, and Firmness

**Figure 1:**
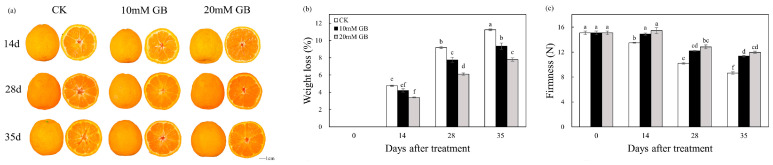
(**a**) Phenotypes control (CK), 10 mM GB (GB-1) treatment, and 20 mM GB (GB-2) treatment ‘Huangguogan’ fruit during postharvest storage. DAT: days after treatment. Changes in (**b**) weight loss and (**c**) firmness of ‘Huangguogan’ fruit in control (CK) and GB-1 and GB-2 treatment groups during postharvest storage. Bars represent the standard deviations of triplicate assays. Different letters show a significant difference (*p* < 0.5).

During the storage period, the phenotypic characteristics of the ‘Huangguogan’ fruit changed. As shown in Figure 1a, there was a change in color of the peel and pulp from light yellow to orange. Additionally, the interstitial space within the fruit’s middle column was observed to expand continuously, but this expansion was effectively inhibited by the application of GB treatment. The weight loss of the fruit exhibited a consistent increase over time (Figure 1b). Treatment with 20 mM GB (GB-2) significantly reduced the weight loss compared to the control (CK) fruit at 14, 28, and 35 DAT. The weight loss of control (CK) was 39.68, 50.25, and 44.20% higher than that of GB-2 treatment at 14, 28, and 35 days after treatment (DAT), respectively. Moreover, there was a decreasing trend in the firmness of the pericarp of the fruit, whereas the control (CK) group exhibited a more rapid decrease in firmness compared to the GB-treated fruit (Figure 1c). While the 10 mM GB (GB-1) treatment led to a notable inhibition of firmness loss, the GB-2 treatment demonstrated an even better effectiveness. At the end of storage, the firmness of GB-treated fruit at 10 and 20 mM was 31.66 and 38.61% higher than that of control (CK) fruit.

### 2.2. Total Soluble Solid, Titratable Acid, and Ascorbic Acid

**Figure 2:**
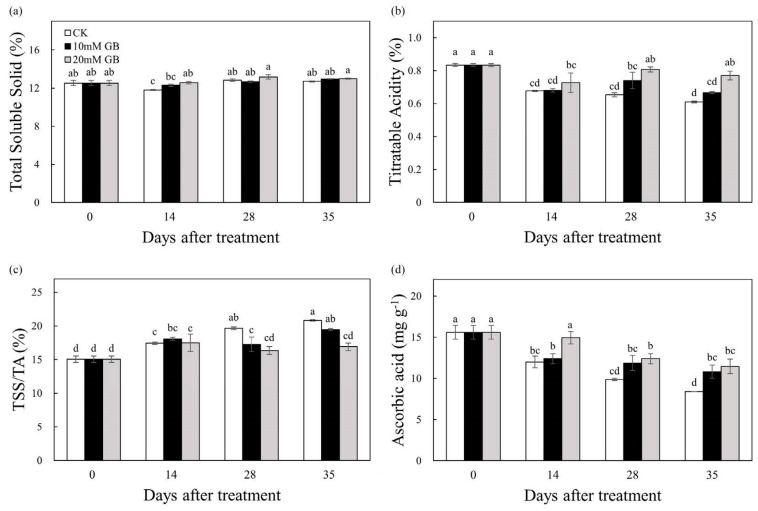
Changes in (**a**) total soluble solids, (**b**) titratable acid, (**c**) TSS/TA, and (**d**) ascorbic acid content of ‘Huangguogan’ fruit in control (CK) and GB-1 and GB-2 treatment groups during postharvest storage. Bars represent the standard deviations of triplicate assays. Different letters show a significant difference (*p* < 0.5).

The total soluble solid (TSS) in the fruit treated with GB-2 showed a slight increase until 28 DAT, after which it remained stable. In comparison to the control (CK) fruit, the GB-2-treated fruit exhibited 6.78% higher TSS levels at 14 DAT (Figure 2a). The TA content gradually decreased during storage, regardless of the different treatments (Figure 2b). However, GB treatment delayed this decrease and maintained it at a higher level compared to the control (CK). The ratio of TSS to TA was influenced by all treatments at 28 and 35 DAT. The control (CK) fruit showed an increasing trend in the TSS/TA ratio during storage, with a significantly higher ratio compared to the GB-2-treated fruit at 28 and 35 DAT (Figure 2c). The ascorbic acid content decreased in all fruit during the storage period (Figure 2d). However, the GB-2-treated fruit consistently had the highest ascorbic acid content 14 DAT, and the content of GB-2-treated fruit was 24.44, 25.68, and 36.51% higher compared to control (CK) fruit at 14, 28 and 35 DAT, respectively.

### 2.3. Organic Acids

**Figure 3:**
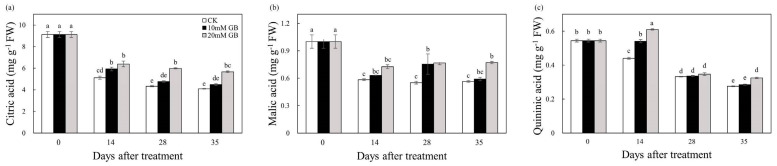
Changes in (**a**) citric acid, (**b**) malic acid, and (**c**) quininic acid of ‘Huangguogan’ fruit in control (CK) and GB-1 and GB-2 treatment groups during postharvest storage. Bars represent the standard deviations of triplicate assays. Different letters show a significant difference (*p* < 0.5).

In this study, the content of citric, malic, and quininic acid was determined. The content of citric acid gradually declined during the storage period (Figure 3a). In the control (CK) group, the citric acid content decreased by 54.95% during storage, while the citric acid content in GB-1- and GB-2-treated fruit decreased by 50.55 and 37.36%, respectively. GB treatment significantly inhibited the decrease. The malic acid content decreased before 14 DAT, then exhibited a slight increase until 28 DAT in GB-1-treated fruit (Figure 3b). Conversely, the content in the control (CK) fruit continuously decreased, and was significantly lower compared to GB-2-treated fruit at 28 and 35 DAT. The content of quininic acid steadily decreased in the control (CK) fruit, while it increased in the GB-2 treatment fruit before 14 DAT, and then declined (Figure 3c). The treatment appeared to have an inhibitory effect on this decrease. At the last sampling time, the quininic acid content in GB-2 treatment fruit was significantly higher than that in GB-1 treatment and control (CK) fruit. The findings indicated that GB-2 treatment was more effective than GB-1 treatment.

### 2.4. MDA and H_2_O_2_

**Figure 4:**
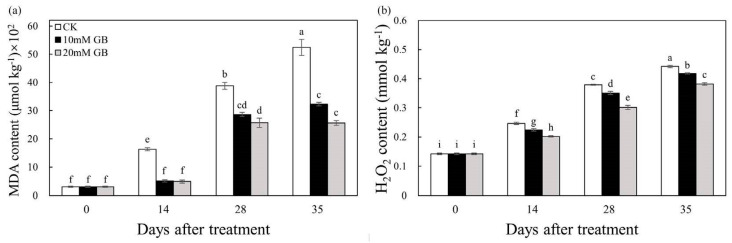
Changes in (**a**) MDA and (**b**) H_2_O_2_ of ‘Huangguogan’ fruit in control (CK) and GB-1 and GB-2 treatment groups during postharvest storage. Bars represent the standard deviations of triplicate assays. Different letters show a significant difference (*p <* 0.5).

To investigate the extent of peroxidation in lipid membranes, our study measured the content of MDA and H_2_O_2_. Over the course of storage, both the GB-treated and control (CK) fruit showed an increasing trend in MDA and H_2_O_2_ contents, whereas the application of GB significantly suppressed the accumulation of these substances throughout the entire storage period. It was observed that GB-1 treatment reduced the accumulation of MDA and H_2_O_2_, but GB-2 treatment was found to be more effective in this regard. Figure 4a illustrates that the control (CK) fruit exhibited an increasing trend in MDA accumulation throughout storage, with levels 33.78% and 55.17% higher than those in GB-2-treated fruit at 28 and 35 DAT, respectively. Furthermore, the highest level of H_2_O_2_ was observed in the control (CK) fruit, and the lowest level was found in GB-2-treated fruit (Figure 4b). Specifically, the H_2_O_2_ levels in the control (CK) fruit were 18.10%, 20.58%, and 13.65% higher than that of GB-2 treatment at 14, 28, and 35 DAT, respectively.

### 2.5. Total Phenolic Content, Total Flavonoid Content, and Antioxidant Enzyme Activity

**Figure 5:**
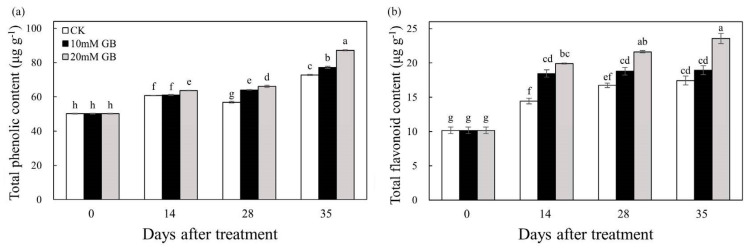
Changes in (**a**) total phenolic and (**b**) total flavonoids of ‘Huangguogan’ fruit in control (CK) and GB-1 and GB-2 treatment groups during postharvest storage. Bars represent the standard deviations of triplicate assays. Different letters show a significant difference (*p* < 0.5).

To assess the antioxidant capacity of ‘Huangguogan’ fruit, we measured the total phenolic and total flavonoid contents. The results presented in Figure 5a illustrate that the total phenolic content of GB-treated fruit exhibited a gradual increase from 0 to 35 DAT. Furthermore, the total phenolic accumulation in fruit treated with GB-2 was significantly higher compared to the control (CK) at 14, 28 and 35 DAT. Similarly, the detection of total flavonoid content (Figure 5b) revealed a rapid increase from 0 to 14 DAT, followed by a slight increase until the end of storage. Notably, the control (CK) fruit had the lowest total flavonoid content, whereas the fruit treated with GB-2 consistently recorded the highest content throughout the entire storage period. Moreover, the total flavonoid contents in GB-2-treated fruit were 37.87%, 29.08%, and 35.29% higher than that in the control (CK) at 14, 28, and 35 DAT, respectively.

**Figure 6:**
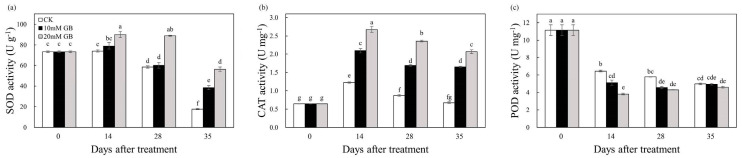
Changes in (**a**) SOD activity, (**b**) CAT activity, and (**c**) POD activity of ‘Huangguogan’ fruit in control (CK) and GB-1 and GB-2 treatment groups during postharvest storage. Bars represent the standard deviations of triplicate assays. Different letters show a significant difference (*p* < 0.5).

The SOD activity was significantly increased by GB-2 treatment compared to the GB-1-treated fruit and control (CK) 14 DAT (Figure 6a). Initially, the SOD activity increased gradually before 14 DAT, after which it declined. However, the GB-2 treatment effectively suppressed this decrease, and the SOD activities in the fruit of control (CK) were 17.95, 34.22, and 68.49% lower than that of GB-2 treatment at 14, 28, and 35 DAT. A similar trend was observed in the CAT activity (Figure 6b). The control (CK) group exhibited the lowest CAT activity, whereas the GB-2-treated fruit had the highest, indicating that GB treatment significantly increased the CAT activity. The POD activity initially decreased rapidly and then remained relatively stable in the GB-treated fruit, while the control (CK) fruit showed a decline from 14 to 35 DAT (Figure 6c). Interestingly, the control (CK) fruit had the highest POD activity throughout the storage period.

### 2.6. Transcriptome Analysis and Enrichment of DEGs

To investigate the potential mechanism of the GB effect in postharvest storage, we conducted a transcriptome analysis on control (CK) and GB-2-treated fruit samples with three replicates at 28 DAT. The RNA from both groups was extracted and subjected to data quality control (CK). A total of 37.02 Gb of clean bases was obtained, with an average number of 41,264,183 clean reads. Furthermore, 90.33% to 92.28% of the clean reads were successfully mapped to the *Citrus mangshanensis* reference genome, with a Q30 value of at least 94.06% and a GC content ranging from 44.30% to 44.43%.

**Figure 7:**
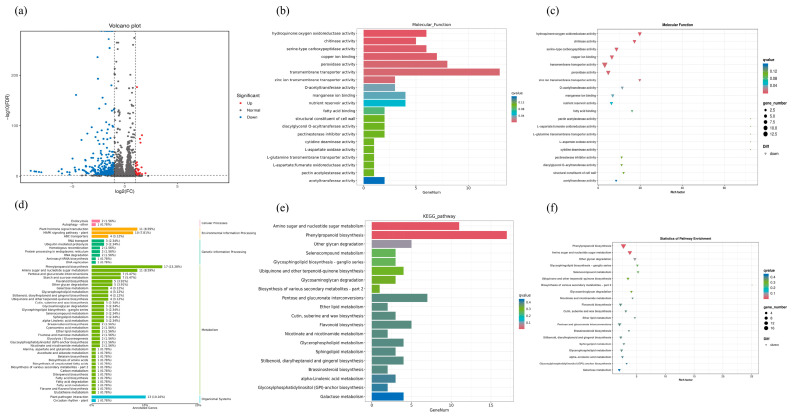
(**a**) Volcano plots of DEGs in control (CK) vs. GB-2 treatment. The red, blue, and gray dots represent up-regulated, down-regulated, and non-significant DEGs, respectively. The horizontal axis represents the fold change in gene expression levels, and the vertical axis represents the significance of differences. (**b**,**c**) GO enrichment analysis of down-regulated DEGs in molecular function of control (CK) vs. GB-2 treatment. (**d**–**f**) KEGG pathway enrichment analysis of down-regulated DEGs of control (CK) vs. GB-2 treatment.

**Figure A1:**
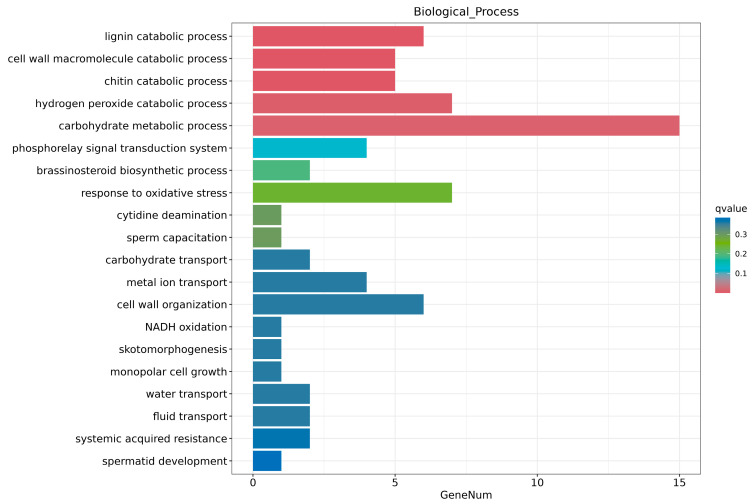
GO enrichment analysis of down-regulated DEGs in the biological process of control (CK) vs. GB-2 treatment.

**Figure A2:**
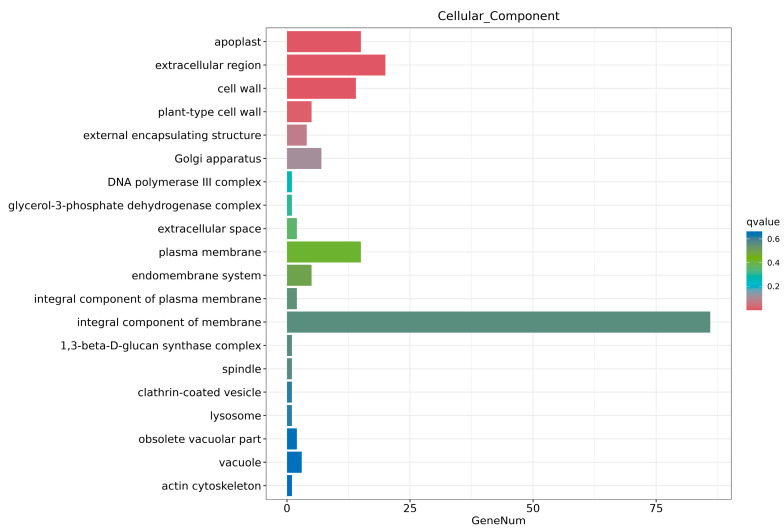
GO enrichment analysis of down-regulated DEGs in the cellular component of control (CK) vs. GB-2 treatment.

DEGs were identified using a stringent criterion of |log2 FoldChange| ≥ 1 and *p*-value < 0.05. The results, as depicted in Figure 7a, revealed a total of 391 DEGs between GB-2 treatment and the control (CK) group. Of these DEGs, 342 genes were found to be up-regulated while 49 genes were down-regulated in the GB-2 treatment group compared to the control (CK) group. Furthermore, the DEGs showed significant enrichment in various biological processes, cellular components, and molecular functions based on the Gene Ontology (GO) database. Notably, the GO category of biological process exhibited enrichment in carbohydrate metabolic and hydrogen peroxide catabolic processes (Figure A1). In terms of cellular components, DEGs were primarily associated with integral components of the membrane and nucleus (Figure A2). Additionally, the molecular function analysis indicated enrichments of DEGs in oxygen oxidoreductase activity and peroxidase activity (Figure 7b,c).

DEGs were aligned with the KEGG database reference in the control (CK) vs. GB-2 treatment comparison (Figure 7d). The result revealed that the DEGs were significantly enriched in various aspects such as cellular processes, environmental information processing, genetic information processing, metabolism, and organismal systems. Notably, the phenylpropanoid biosynthesis pathway exhibited the highest level of enrichment, with the largest number of DEGs. Additionally, enrichments of down-regulated DEGs were detected in amino sugar and nucleotide sugar metabolism, pentose and glucuronate interconversions, and flavonoid biosynthesis in the control (CK) compared to GB-2 treatment group (Figure 7e,f).

## 3. Discussion

Long-term storage leads to a decline in bioactive compound content and antioxidant activity, ultimately impacting the quality of the fruit. Consequently, postharvest fruit preservation has become a challenge for the industry. Previous research has reported postharvest preservation focused on plant growth regulators, such as putrescine [23], cytokinin [24], and γ-aminobutyric acid [25], due to their non-toxic and residue-free properties. Recently, an increasing number of studies has been reported in which GB was applied to horticulture crops in postharvest storage [26,27,28]. Our study revealed that exogenous application of GB significantly retarded the senescence process of ‘Huangguogan’ fruit, thereby positively effecting its quality and antioxidant activity.

Fruit quality, which encompasses attributes such as sugar, acidity moisture, and firmness, plays a crucial role in determining the acceptability of citrus fruit. Soluble sugar is the main constituent of TSS in citrus fruit. During the storage period, the TSS content showed a slight increase (Figure 2a). Roghayeh et al. [19] discussed that the increase in TSS content in postharvest fruit is attributed to the hydrolysis of cell wall polysaccharides. The TA of ‘Huangguogan’ fruit decreased over time, but the TA content of fruit treated with GB was significantly higher than that of the control (CK) fruit, indicating that GB inhibited the decline in TA content (Figure 2b). Organic acids are metabolized in the mitochondria through the tricarboxylic acid cycle, which is related to respiratory metabolism. Citric, malic, and quininic acids were detected in all samples (Figure 3), with both GB-1- and GB-2-treated fruit showing higher levels than the control (CK), suggesting that GB effectively suppressed the degradation in organic acids. The decrease in organic acid content is a result of sugar and phenolic compound synthesis [22]. Ma et al. [29] reported that a highly acidic mutant of ‘Bingtang’ sweet orange has better storage performance than low-acid mutants, manifesting the importance of organic acids in the preservation of fruit after harvest. In our study, GB treatment significantly maintained the organic acid content; therefore, it is inferred that GB treatment enhanced the storability of ‘Huangguogan’ fruit. Further investigation is warranted to elucidate the underlying mechanism by which GB influences the metabolic processes of organic acids. Water diffusion, which is the primary cause of weight loss, is a major contributor to postharvest deterioration [30]. This study demonstrated that after treatment with GB, the ‘Hunagguogan’ fruit exhibited a significant reduction in weight loss, particularly in the GB-2-treated fruit (Figure 1b). Similar effects have been observed with GB treatment in enhancing water retention capacity of pears [15] and plums [19]. It should be noted that water loss can lead to increased oxidation in citrus fruits, resulting in the loss of ascorbic acid [31]. GB treatment effectively reduced the weight loss and maintained a relatively high level of water, thereby alleviating physiological disorders caused by oxidant stress. The firmness of citrus fruit is a crucial parameter that influences its market acceptability. GB acts as an osmotic regulator and effectively maintains the structure of biological macromolecules [32], thereby protecting the stability of cell membranes [33]. In this study, the GB-treated fruit exhibited a significantly lower firmness loss compared to the control (CK) fruit (Figure 1c). Moreover, there were enrichments in DEG’s integral component of the membrane and cell wall, shown in the GO analysis (Figure A1), which inferred that application of GB has an influence on the membrane integrity of ‘Huangguogan’ fruit during postharvest storage. Zhang et al. [14] reported that GB treatment delayed the firmness loss of winter jujube fruit by inhibiting the activity of cell-wall-modifying enzymes. Additionally, Zhang et al. [34] reported that firmness was influenced by the degree of lipid peroxidation, which was related to the accumulation of MDA and H_2_O_2_.

The maintenance of fruit quality not only affects the sensory characteristics of the fruit but also safeguards it against physiological disorders under various stress. During the process of long-term storage, the fruit undergoes oxidative stress, which leads to physiological disorders and damages the integrity of the membrane by generating ROS such as H_2_O_2_, singlet oxygen, and hydroxyl radicals [35]. The final product of cellular oxidation reactions is MDA, and its level serves as an indicator of lipid peroxidation. In our study, the MDA content increased in all samples during storage. However, GB treatment notably suppressed the accumulation of MDA compared to the control (CK) group, particularly in GB-2-treated fruit (Figure 4a). Similar findings have been demonstrated in winter jujube fruit [14] and ‘Nanguo’ pears [15], highlighting that GB alleviated oxidative damage in horticultural crop cells by scavenging ROS generated in metabolism of cells during storage. It is inferred that GB treatment effectively retains a high concentration of endogenous antioxidant components by up-regulating gene expression. The accumulation of MDA is primarily caused by the excessive production of H_2_O_2_ through mitochondrial respiration. Its long half-life allows for the continuous destruction of cells in the fruit membrane [36]. In our study, the increased content of H_2_O_2_ indicated a significant acceleration in oxidant stress (Figure 4b). However, GB treatment reduced H_2_O_2_ accumulation, and similar results were reported in winter jujube fruits [14], blood oranges [37], and plum fruits [19]. The accumulation of H_2_O_2_ is influenced by various factors, including storage conditions, fruit species, and antioxidant capacity [38]. Consequently, the reduction in H_2_O_2_ levels is primarily attributed to the presence of antioxidant compounds and enzymes [19]. The antioxidant defense system, which comprises both enzymatic and non-enzymatic antioxidants, plays a crucial role in controlling the senescence of postharvest fruit by effectively scavenging excessive ROS and maintaining redox homeostasis [39]. A transcriptome analysis revealed enrichments in the antioxidant defense system, as indicated by GO and KEGG pathway analyses (Figure 7b,e). Specifically, genes related to oxygen oxidoreductase activity were found to be enriched and significantly up-regulated in the GB-2 treatment group compared to the control (CK). Enzymatic antioxidants such as SOD, POD, and CAT, along with non-enzymatic antioxidants like ascorbic acid, phenols, and flavonoids, constitute the defense system [38]. The combined action of these two systems influences the level of ROS in postharvest fruit. For instance, SOD converts superoxide anion radicals into H_2_O_2_ and O_2_, while ascorbic acid, phenols, and flavonoids decompose H_2_O_2_ into H_2_O and O_2_. Furthermore, GB exerts its antioxidant function by regulating cell osmotic pressure, preserving enzymatic structures and functions, and enhancing the stability of antioxidant enzymes [7]. In our study, GB treatment effectively increased SOD and CAT activities (Figure 6). Additionally, GB not only increased the activity of enzymes but also up-regulated the gene expression of these enzymes in winter jujube fruit [14] and ‘Nanguo’ pears [15]. Based on these studies, it can be concluded that GB treatment effectively activated the ability to resist oxidative stress through gene-level regulation. Moreover, GB significantly maintained the levels of ascorbic acid and total phenolic compounds simultaneously (Figure 5). A similar result has been observed in bananas [28], mushrooms [40], and zucchini fruits [35]. We measured the total flavonoid content, and the results showed that the content in GB-2-treated fruit was significantly higher compared to the control (CK). A KEGG analysis revealed an enrichment in flavonoid biosynthesis, which involves a complex process catalyzed by numerous enzymes [41]. However, there is limited research on the mechanism of GB action in flavonoid biosynthesis, and further studies should focus on the transcriptional regulation of this mechanism. As a result, the activities of antioxidant enzymes and other antioxidants such as ascorbic acid, phenols, and flavonoids were enhanced by GB treatment, thereby alleviating lipid peroxidation and delaying fruit senescence.

## 4. Materials and Methods

### 4.1. Plant Materials and Treatment

‘Huangguogan’ fruit was picked at the commercial maturity stage from a commercial citrus orchard in Shimian County, Yaan City, Sichuan Province, in 2022. The fruit selected was of uniform size, without disease, pests, and mechanical injury, and was transported to laboratory in Chengdu within 5 h.

A total of 900 fruits were randomly and equally divided into three groups, after rinsing with tap water. One group was dipped into 10 mM or 20 mM GB (Shanghai Macklin Biochemical Co., Ltd. Shanghai, China) that was dissolved in 40 L of distilled water for 5 min at room temperature. The control (CK) group was dipped into distilled water for 5 min at room temperature. After treatment, all fruits were air-dried, then samples were put into a polyethylene bag (40 cm × 30 cm) and stored at room temperature and 60% relative humidity. Each group, containing three biological replicates, was taken to measure the fruit quality and antioxidant enzyme activity at 0, 14, 28, and 35 DAT, respectively. There were 20 fruits per replicate.

### 4.2. Weight Loss and Firmness

The percentage of fruit weight loss was reported by weighing the initial weight (fresh weight) before storage and the final weight (fresh weight) at each sampling time. Fruit firmness was measured using a TMS-Pilot Precision texture analyzer (FTC, Sterling, VA, USA). The initial force, range of force, puncture speed, and puncture distance were set to 0.75 N, 450 N, 60 mm min^−1^, and 15 mm, respectively [21].

### 4.3. Total Soluble Solid, Titratable Acidity, and Ascorbic Acid

The TSS and TA contents (fresh weight) were measured by an integrated sugar and acid machine (Pocket PAL-BXIACID1; ATAGO, Tokyo, Japan). The machine was zeroed by measuring distilled water, then 300 μL citrus juice was added to determine the TSS content. After finishing TSS detection, the machine was quickly wiped with absorbent paper and zeroed again with distilled water. A total of 1 mL of juice was added into a beaker and diluted to 50 mL with distilled water. The machine was switched to acid detection mode, then TA detection was carried out. The ascorbic acid content (fresh weight) was detected by the method of Kelebek and Selli with slight modifications [42]. A total of 5 mL of juice was diluted to 50 mL with 1% oxalic acid, then 5 mL of the dilution was pipetted into a 50 mL triangular flask and titrated with a 2,6-dichlorophenol indophenol dye solution until the color of dilution was continuously pink. The ascorbic acid content was expressed as mg g^−1^.

### 4.4. Organic Acids Detection

The organic acid components (fresh weight) of the pulp were detected by high performance liquid chromatography (LC-1260; Agilent Technologies, Sacramento, CA, USA). Detection of the content of organic acids followed the method described by Wang et al. [43] with slight modifications. The pulp samples were weighed to 0.5 g, ground with pre-chilled 3 mL of 0.2% metaphosphoric acid, fixed to 6 mL, and centrifuged at 4 °C for 15 min at 12,000 r min^−1^. The supernatant was extracted and filtered through a 0.22 µm aqueous membrane for the determination of organic acid components [21]. The content of organic acids was determined by the standard curve for each substance.

### 4.5. Total Phenolic and Total Flavonoids Contents and Antioxidant Enzymes

The total phenolic content (fresh weight) was measured using the Folin–Ciocalteu method [44]. The content was extracted from 0.5 g of frozen tissue with 2.5 mL of methanol and centrifuged at 4 °C for 20 min at 10,000 r min^−1^. The supernatant was mixed with 1 mL of 2% sodium carbonate solution and reacted for 5 min at room temperature. Then, 0.25 mL of Folin–Ciocalteu phenol reagent (50%) was added and reacted for 30 min without light. The absorbance was measured at 650 nm by a spectrophotometer (FI-01620, Thermo Fisher Scientific Oy, Vantaa, Finland) and standard curves were constructed with a gallic acid aqueous solution. The total phenolic content was expressed as μg g^−1^.

The level of total flavonoids (fresh weight) was estimated by the following method described by Baswa et al. [45] with slight modifications. The total flavonoid content was extracted from 0.3 g of frozen tissue with 3 mL of a pre-chilled 80% methanol aqueous solution centrifuged at 4 °C for 15 min at 12,000 r min^−1^. The supernatant was mixed with 0.5 mL of 10% aluminum chloride, then the mixture was incubated for 30 min. The absorbance was read at 500 nm wavelength and the content of total flavonoids was determined by the standard curve and expressed as μg g^−1^.

The method of SOD, POD, and CAT activity (fresh weight) detection followed the method of Zhang et al. [17] with minor modifications. SOD and POD were extracted from 0.5 g of frozen tissue with 5 mL of pre-chilled 50 mM sodium phosphate buffer (pH 7.8). After mixing, the homogenate was centrifuged at 4 °C for 20 min at 12,000 r min^−1^. The supernatant was extracted to determine SOD and POD activity using a spectrophotometer (FI-01620, Thermo Fisher Scientific Oy, Vantaa, Finland) at 560 and 470 nm, respectively. One unit of SOD activity is defined as the amount of enzyme causing half the maximum inhibition of nitro blue tetrazolium (NBT) reduction. CAT was extracted from 0.5 g of frozen tissue with 5 mL of pre-chilled 50 mM sodium phosphate buffer (pH 7.5) which contained 0.1 mM ethylene diamine tetra acetic acid (EDTA). The mixture centrifugation method was the same as above. One unit of CAT is defined as the amount of enzyme that decomposes 1 μmol of H_2_O_2_ every minute. The unit of SOD activity was expressed on a fresh weight basis as U g^−1^, while POD and CAT activities were expressed as U mg^−1^.

### 4.6. Malondialdehyde (MDA) and Hydrogen Peroxide (H_2_O_2_) Content

The MDA content (fresh weight) was detected according to the method described by Sun et al. [15] with slight modifications. An amount of 0.5 g of frozen pericarp tissue was homogenized in 5 mL of 10% trichloroacetic acid (TCA) and centrifuged at 10,000 r min^−1^ for 10 min at 4 °C. A total of 1 mL of supernatant was added to 2 mL of 0.67% thiobarbituric acid and boiled for 20 min at 100 °C. After cooling, the absorbance of the supernatant was detected at 450, 532, and 600 nm by a spectrophotometer (FI-01620, Thermo Fisher Scientific Oy, Vantaa, Finland). The MAD content was expressed as mmol kg^−1^. The H_2_O_2_ content (fresh weight) was determined according to the method described by Fariborz Habibi et al. [37]. An amount of 1 g of frozen tissue was homogenized with 2 mL of 1% trichloroacetic acid and centrifuged at 12,000 r min^−1^ for 15 min at 4 °C. Additionally, 250 μL of 100 mM phosphate buffer (pH 7.0) and 500 μL of potassium iodide (KI) were added into 250 μL of the supernatant. The absorbance of the supernatant was detected at 390 nm by a spectrophotometer (FI-01620, Thermo Fisher Scientific Oy, Vantaa, Finland). A standard diagram of hydrogen peroxide was drawn to determine the H_2_O_2_ content which was expressed in mmol kg^−1^.

### 4.7. Transcriptome Analysis

The fruit RNA of the GB-2 treatment group and control (CK) at 28 days after treatment was extracted, and each group included three biological replicates. RNA purity was assessed using a Nanodrop2000 (Thermo Fisher Scientific, Vantaa, Finland) and integrity was detected by an Agient2100, LabChip GX (Agient Technologies, Santa Clara, CA, USA). cDNA library construction and high-through sequencing were commissioned by Biomarker Technologies Co., Ltd. (Beijing, China). The total RNA was used as input material for RNA sample preparation. Through combination of RNA and paramagnetic particles, random disruption, reverse transcription amplification, and total RNA, which satisfies the standard of testing, double-stranded DNA was obtained and synthesized, followed by performance of the polymerase chain reaction (PCR) [21]. Sequencing libraries were generated via PCR and library quality and sequenced on an Illumina Novaseq platform. In order to obtain clean reads, reads which contained adapter and poly-N and low-quality reads were removed from the raw data and the Q20, Q30, and GC contents of the clean data were calculated. All generated clean reads were mapped to the ‘mangshanyegan’ citrus reference genome. Genes with |log2 FC| ≥ 1 and *p* < 0.05 were considered as differentially expressed genes (DEGs) in a comparative analysis. Enrichment of DEGs was analyzed by Gene Ontology (GO), in which *p* < 0.05 was considered a significant enrichment. The enriched pathways were searched for using the Kyoto Encyclopedia of Genes and Genomes (KEGG) pathway analysis [46].

### 4.8. Statistical Analysis

The experiments were repeated three times and analyzed using multiple comparisons (LSD) with IBM SPSS Statistics 23.0 (IBM, Armonk, NY, USA). The analytical variance of data was presented as the means ± standard errors compared using a Tukey-HSD test, and the statistical significance was set at *p* < 0.05.

## 5. Conclusions

In conclusion, this study revealed the effect of GB treatment on the quality and antioxidant activity of ‘Huangguogan’ fruit postharvest. GB treatment exhibited the ability to preserve the flavor of ‘Huangguogan’ fruit by maintaining the TSS and organic acid contents. Additionally, after GB treatment, the weight and firmness loss decreased, resulting in a delay in the softening of the fruit. Moreover, the enrichments of DEGs in oxygen oxidoreductase activity evidenced that GB treatment stimulated the antioxidant system and effectively eliminated ROS, thereby mitigating lipid peroxidation. The findings indicated that a concentration of 20 mM GB proved to be more efficacious compared to other treatments, thus providing a valuable postharvest preservation strategy for this particular citrus variety.

## Data Availability

Data are contained within the article.
